# Supplementary material for: Complexome profiling on the Chlamydomonas lpa2 mutant reveals insights into PSII biogenesis and new PSII associated proteins
Source: J Exp Bot. 2021 Aug 26;73(1):245–62. doi: 10.1093/jxb/erab390 (PMC8730698; doi:10.1093/jxb/erab390)
Supplement: erab390_suppl_Supplementary_Dataset_S1 [file erab390_suppl_supplementary_dataset_s1.zip › Supplemental Dataset 1 - Excel List and all profiles/plots/LEU3_Cre07.g325400.html]

Trivial name: LEU3  
  
Euclidean distance: 2426.51  
Mean Intensity (WT): 81.66  
Mean Intensity (Mut): 51.25  
Distance: 29.72  
  
MapMan: secondary metabolism.sulfur-containing.glucosinolates.synthesis.aliphatic.methylthioalkylmalate dehydrogenase (MAM-DH);amino acid metabolism.synthesis.branched chain group.leucine specific.3-isopropylmalate dehydrogenase  
  
p value of intensity sums Welch test: 0.7711
